# HIV Monoclonal Antibodies: A New Opportunity to Further Reduce Mother-to-Child HIV Transmission

**DOI:** 10.1371/journal.pmed.1001616

**Published:** 2014-04-08

**Authors:** Yegor Voronin, Lynne M. Mofenson, Coleen K. Cunningham, Mary G. Fowler, Pontiano Kaleebu, Elizabeth J. McFarland, Jeffrey T. Safrit, Barney S. Graham, William Snow

**Affiliations:** 1 Global HIV Vaccine Enterprise, New York, New York, United States of America; 2 Eunice Kennedy Shriver National Institute of Child Health and Human Development, National Institutes of Health, Rockville, Maryland, United States of America; 3 Duke University Medical Center, Durham, North Carolina, United States of America; 4 Johns Hopkins University School of Medicine, Baltimore, Maryland, United States of America; 5 Medical Research Council/Uganda Virus Research Institute Uganda Research Unit on AIDS, Entebbe, Uganda; 6 University of Colorado School of Medicine, Denver, Colorado, United States of America; 7 Elizabeth Glaser Pediatric AIDS Foundation, Los Angeles, California, United States of America; 8 Vaccine Research Center, National Institute of Allergy and Infectious Diseases, National Institutes of Health, Bethesda, Maryland, United States of America

## Abstract

Yegor Voronin and colleagues explore how monoclonal antibodies against HIV could provide a new opportunity to further reduce mother-to-child transmission of HIV and propose that new interventions should consider issues related to implementation, feasibility, and access.

*Please see later in the article for the Editors' Summary*

**Summary Points**

- In 2012, there were 260,000 new pediatric HIV infections worldwide, and it is unlikely that the goal of global elimination set by UNAIDS for 2015 will be met with current antiretroviral interventions alone.
- Researchers have recently isolated and characterized broadly neutralizing antibodies against HIV-1, opening the potential avenue for passive immunization as a complement to standard therapy.
- Populations at the highest risk for MTCT, and, therefore more likely to benefit from new interventions, include women presenting late in pregnancy and/or lacking antenatal care but it is difficult to enroll and retain such women in trials.
- The target product profile of new interventions should consider issues of implementation feasibility and access in the context of local health systems, many of which are resource-constrained.
- A proposed passive immunization trial with the monoclonal antibody VRC01 serves as a useful case study to explore the conduct of trials in infants exposed to HIV-1 via breastfeeding.

## State of HIV MTCT Prevention

With the dramatic progress in effective interventions to prevent mother-to-child HIV transmission (PMTCT), new pediatric HIV infections have become rare in high-income settings. In the last five years, PMTCT interventions have been implemented and have undergone rapid scale-up in low-resource settings, leading UNAIDS to set a new goal to virtually eliminate new pediatric HIV infections by 2015 [1]. Virtual elimination has been defined as a 90% reduction in mother-to-child transmission (MTCT) from 2009 levels, to <40,000 new infections annually and an overall transmission rate of <5% in breastfeeding populations. However, significant implementation challenges remain in the 21 priority countries, making it unlikely that the goal will be met with the existing interventions alone [2].

The transplacental transfer of maternal antibodies to infants protects children from infectious pathogens until immunological maturity is sufficient to produce and regulate effective immune responses. Immunoglobulin transfer continues after birth through breastfeeding, which also provides essential nutrients that are not otherwise available. Unfortunately, during chronic HIV infection the antibodies present in the infected host can generally neutralize virus from three to six months earlier [3], but are not able to neutralize contemporaneous circulating strains. Thus, the antibodies present in the serum of HIV-infected mothers are not sufficient to prevent infection from viruses to which infants are exposed during the intrapartum period and through breast milk. In breastfeeding infants born to HIV-1-infected mothers, overall MTCT can be as high as 40% with prolonged breastfeeding in the absence of antiretroviral (ARV) prophylaxis [4].

**Figure 1:**
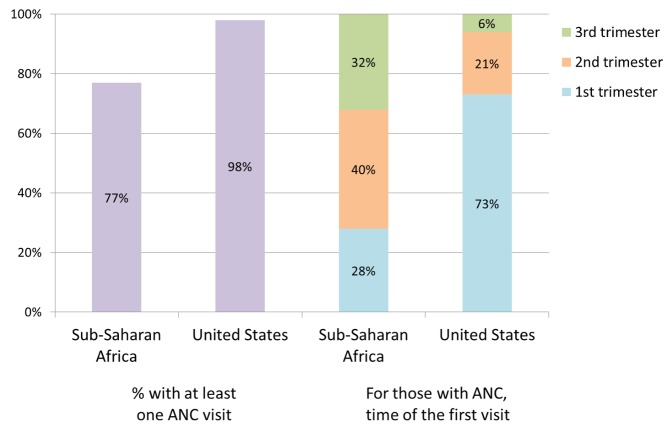
While 77% of women in sub-Saharan Africa have at least one antenatal care (ANC) visit, most are not seen until the second or third trimester [34]. In contrast, the majority of women in the United States access antenatal care during the first trimester [35].

Optimal prevention requires identification of maternal HIV infection early in pregnancy with prompt initiation of ARV therapy. Studies have demonstrated that initiation of therapy later than 13 weeks before delivery is associated with increased risk of MTCT [5]. However, women in low-resource countries may miss opportunities to reduce transmission due to missed HIV screening in antenatal settings, delivery outside of formal medical settings, HIV infection during pregnancy and breastfeeding, and the need to extend breastfeeding to provide the infant with the best overall chance of survival (Figure 1).

While formula feeding is recommended for HIV-infected mothers in industrialized countries, breastfeeding is the cornerstone of infant survival in many low-resource countries. In such settings, the World Health Organization (WHO) recommends that HIV-infected mothers should breastfeed for 12 months with concurrent infant or maternal ARV prophylaxis to reduce transmission risk [2]. However, new reports suggest that weaning prior to age 18 months is associated with elevated morbidity and mortality among HIV-exposed, uninfected children even in clinical trial settings [4]. Additionally, the use of ARV prophylaxis by mother or infant during breastfeeding can reduce but does not eliminate transmission risk and relies on strict adherence to daily drug administration. Breakthrough infections at rates as high as 2–5% by age six months and 6% by age 12 months have been observed in breastfeeding infants of HIV-infected mothers who have been provided with triple ARV drug therapy during pregnancy and breastfeeding [4],[6].

Although ARV prophylaxis significantly reduces MTCT, effective implementation is complicated by the need for prolonged drug administration and adherence, potential toxicities leading to continued monitoring requirements, potential for drug resistance, and inadequate health care infrastructure. Adherence to therapy during the postpartum period has been particularly problematic for women [7],[8]. Since 2009, there has been a 38% decrease in new pediatric HIV-1 infections across the 21 countries in sub-Saharan Africa that account for 90% of all new pediatric infections. However, there were still 210,000 new pediatric infections in these countries in 2012, with an estimated overall transmission rate of 17% (15–20%). Approximately 40–50% of these infections were acquired through breastfeeding [2].

Thus, it appears unlikely that the goal of global elimination will be met with current ARV interventions alone, and continued investigation of preventive interventions to reduce MTCT, including maternal and/or infant passive/active immunization, remains important.

## Monoclonal Antibodies Present New Opportunities

Starting in 2008, a technological revolution has taken place in HIV antibody research. Development of next-generation sequencing, advances in *in vitro* B cell clonal amplification, and high-throughput neutralization assays allow investigators to study the diversity of antibody responses in HIV-infected subjects and to identify antibodies with long-sought properties. Using RT-PCR, both the heavy and light chain immunoglobulin (Ig) genes can be amplified from single B cells and cloned into expression vectors [9]. These techniques allow large-scale isolation and synthetic production of human monoclonal antibodies (mAb) by transfection of producer cells *in vitro*. As a result, the field that had identified only four broadly neutralizing antibodies (bNAbs) in the past 25 years has suddenly found dozens of new bNAbs, some of which were two orders of magnitude more potent, with much greater breadth than their predecessors [10]–[13]. Most of the HIV-1-specific bNAbs found to date have unusual properties including long heavy chain CDR3 loops, extensive somatic hypermutation, and glycan dependence, which may explain why it has been hard to induce bNAbs by vaccination and why the neutralizing antibody response to natural infection lags behind the evolution of neutralization-resistant genetic variants [14].

Whether the newly discovered antibodies could be used to treat or prevent HIV-1 infection is a question that is being actively explored (Box 1) [15]. Historically, passively administered antibodies have been shown to have an excellent safety profile and to protect against multiple viruses, including cytomegalovirus, varicella zoster virus, poliovirus, hepatitis A and B viruses, measles virus, Junin virus, rabies virus, and respiratory syncytial virus (RSV). Polyclonal immunoglobulins have also been shown to be effective in preventing perinatal transmission of several viruses. For example, cytomegalovirus hyperimmune globulin administered to pregnant mothers infected with cytomegalovirus during pregnancy was shown to reduce perinatal transmission by 50% [16]. Before the vaccine for hepatitis B was available, the recommended intervention to prevent perinatal hepatitis B transmission was to administer hepatitis B immunoglobulin to all infants born to hepatitis B surface antigen positive mothers at zero, three, and six months after birth [17]; even with active hepatitis B vaccination, a single dose of hepatitis B immunoglobulin continues to be recommended. Administration of varicella zoster hyperimmune globulin to infants born to mothers with varicella around the time of delivery was also shown to reduce the chances of perinatal transmission of varicella zoster virus [18]. Compared to polyclonal immunoglobulin preparations, mAbs are considered to be safer because they are not derived from blood products. Several mAbs are now in clinical development, and two mAbs are currently licensed for use against the microbial pathogens RSV and anthrax [19],[20].

**Box 1. Development of VRC01 mAb for HIV-1 PMTCT**

VRC01 is a human mAb directed to the CD4 binding site of HIV-1 gp120 [13],[30]–[33]. It has broad neutralizing capacity with over 90% of strains from a 200 virus panel neutralized at 50 µg/ml and excellent potency with over 70% of strains neutralized at <1 µg/ml [13]. It appears to effectively neutralize transmitted strains of HIV-1. In Zambian mother-infant pairs in which HIV-1 transmission occurred, VRC01 neutralized 18/23 (78%) isolates from infants, even though only 12/23 (52%) maternal isolates were neutralized [36]. Unlike some other bNAbs, VRC01 has been shown to have no auto-reactivity or tissue cross-reactivity *in vitro*.

In animal model studies, VRC01 has been evaluated in adult and infant macaques. Adults treated with 20 mg/kg of the antibody were consistently protected from all rectal and vaginal challenges with SHIVSF162(P3) (a virus relatively difficult to neutralize) when serum levels were above 40 µg/ml and were partially protected at doses of 5 mg/kg. Protection from SHIVBAL (a relatively easy to neutralize virus) occurred with doses of less than 1 mg/kg and VRC01 mAb serum levels above 1 µg/ml (personal communication, B. Graham to authors). A small study in seven infant macaques (one-month-old) showed that six animals receiving VRC01 were protected from oral SHIVSF162(P3) challenge (personal communication, N. Haigwood to authors). These doses and the range of mAb levels in serum have been achievable in humans treated with other mAbs.

For these reasons, VRC01 is being considered for evaluation as an adjunct to ARVs in mother-infant pairs with high risk of transmission. Currently, VRC01 has been manufactured at a concentration of 100 mg/ml and phase I pharmacokinetic evaluation in HIV-infected and uninfected adults has begun.

Studies of polyclonal HIV-1 immunoglobulin for PMTCT have not demonstrated efficacy [21],[22]. However, only a small fraction of total IgG in these preparations was HIV-1-specific, and sera were selected based on the presence of antibodies to p24, an internal protein that is not a target for neutralizing antibodies. The bNAbs that are proposed for use in the new studies are known to be effective *in vitro* at concentrations that can be reached in blood after a single injection. They neutralize a diversity of HIV strains, including “transmitter founder” viruses—clones reconstructed from phylogenetic data and representing the individual virions that established infections in the studied individuals. Moreover, recent experiments in macaques have shown that bNAbs can protect against mucosal viral challenge in rhesus macaques [23]. Thus, the new approaches that are based on monoclonals may be expected to be more effective than past attempts with polyclonal sera.

Although ARV prophylaxis is the foundation for PMTCT, active or passive immunization could provide a safe and durable adjunctive intervention to further reduce transmission, particularly during breastfeeding. If efficacious, these approaches have the advantage of being less reliant on patient adherence than interventions requiring daily administration. Adding passive administration of bNAbs to ARV treatment in PMTCT is attractive because of the safety track record for immunoglobulin prophylaxis in general, the long half-life that may cover gaps in ARV adherence, product stability, and the familiarity of practitioners and patients with immunoglobulin treatment to prevent viral diseases. Infants exposed to HIV via their mothers represent a population that may be particularly suited to benefit from passive immunizations because the fact of exposure and duration of exposure can be clearly identified and because the required dose for an infant is smaller than for an adult.

## Bridging Scientific Plausibility and Clinical Practice

The jump from the scientific plausibility of a biomedical intervention to feasible clinical practice is often complex. A January 2013 meeting in Entebbe, Uganda organized by the Global HIV Vaccine Enterprise and others engaged stakeholders around potential challenges in studying and implementing active and/or passive immunization to further reduce MTCT in low-resource settings [24]. A number of key considerations were raised, which are applicable to all efforts to develop additional prevention modalities, either by active or passive immunization.

### The Choice of the Product

The new technologies that bolstered rapid progress in discoveries of human antibodies against HIV-1 are being improved, and new mAbs are being identified. Combinations of antibodies can be used to improve the potency and expand the breadth of viral coverage even further. The choice to develop a particular antibody or a combination of antibodies for clinical research has to take multiple factors into account. Among these are the activity of the product against viruses in the target population, auto-reactivity, administration route, half-life, the ability to manufacture the product in sufficient amounts, and the current stage of product development. Right now, one monoclonal antibody with broad neutralizing activity, VRC01, is slightly more advanced in the development process than other products, and phase I clinical evaluation in adults has begun (see Box 1). However, in the future, the number of potential options will undoubtedly increase and make choices more difficult, requiring consideration of cost∶benefit, risk∶benefit, and timing.

### Clinical Path to Testing Efficacy in Infants

**Figure 2:**
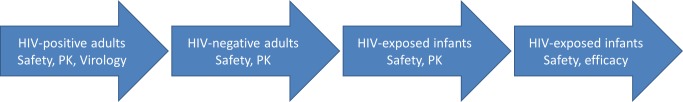
One possible clinical pathway for testing monoclonal antibodies in infants. Safety, pharmacokinetics (PK), and biological effect on virus populations are initially assessed in HIV-positive adults. Dosing is further refined in studies in HIV-negative adults and infants. This information is used to design an efficacy study in HIV-exposed infants as an adjunct to standard ARV treatment and prophylaxis.

The primary objective for passive administration of a monoclonal bNAb to infants would be to prevent infection. The mAb would therefore be given to the infant immediately after delivery with the hope of preventing some intrapartum transmission events and to establish adequate levels of neutralizing activity in the infant's serum to prevent breast milk transmission. The mAb would specifically not be administered to mothers to avoid the possibility of producing neutralization-resistant variants. Before performing these studies in high-risk infants, a series of safety and pharmacokinetic studies will need to be done in adults. Figure 2 outlines one potential sequence of trials that would provide the safety and pharmacokinetic data needed to support a phase IIb efficacy trial, and would also provide logistical data to inform the feasibility analysis being done in parallel as outlined below. Evaluation in HIV-uninfected adults could proceed to testing a small cohort of HIV-exposed infants before determining the dose and schedule for a phase IIb test-of-concept efficacy trial in high-risk infants. Studying the mAb in HIV-infected adults would be informative for two major reasons. First, because some infants may be unknowingly infected at birth, safety data for mAbs in the presence of HIV infection will be needed. Second, the impact on viral replication and pathogenesis could be assessed to indirectly support the primary objective of the prevention trial. Because the typical half-life of a mAb *in vivo* is 21–24 days, the dosing would be monthly and continue until the completion of breastfeeding. Therefore, multi-dose safety data from early phase trials will be needed. These studies would take up to 18 months and require careful planning and coordination with clinical investigators and stakeholders at trial sites, in addition to ongoing communication with and guidance from regulatory agencies.

### Challenges for Conducting Efficacy Trials

Passive immunization is proposed to complement and improve upon the existing PMTCT interventions. Therefore, in clinical trials the mAbs must be tested in combination with local standard of care for HIV prevention. Our estimates show that, with currently observed rates of pediatric HIV-1 infections in infants born to HIV-infected mothers who receive antiretroviral therapy starting either late in pregnancy or postpartum (estimated to be 3–5% based on several clinical trials [25]–[27]), a study would need to enroll 1,000–3,000 mother-infant pairs to be able to detect efficacy of 60% or more for the adjunct intervention. The variability in the required sample size depends on the expected transmission rate, which will be dependent on inclusion criteria that determine the level of transmission risk, and on estimated attrition rate. One of the key populations that would need to be enrolled in the study is women presenting late to antenatal clinics: that is, women with a high risk of transmission even with optimal ARV intervention. Recruitment into the trial will have to be done during the short time between their arrival and delivery, a time when they are particularly vulnerable and may be dealing with the HIV diagnosis. Retaining these women in a trial that requires multiple, regular visits will also be challenging because late presentation is usually caused by socioeconomic disenfranchisement and difficulty accessing medical care.

### Cost Considerations

mAbs are expensive to produce, and it has been argued that the product would not be affordable in resource-limited settings for the population that will need it most [28]. However, any cost-benefit analysis should compare the short-term costs of delivering the prophylaxis with the costs of lifelong treatment and care for an HIV-infected infant. These costs vary widely from one setting to another and will continue to decrease in the future, as both the costs for treatment and for bulk production of antibodies are rapidly improving.

### Feasibility for Integration with Current Standard of Care

The goal of product development should be to seek a target profile that will be feasible with minor changes in the countries' health care infrastructure. Passive immunization would provide protection that that does not depend on strict daily adherence to ARV drugs; thus, passive immunization would be an important adjunct to increase efficacy of current ARV regimens. However, it requires the product to be regularly administered parenterally by qualified medical personnel and, therefore, attention would need to be paid to injection safety, a reliable supply chain, and a plan for medical waste. Required frequency of administration will be a critical factor in product development. Pharmacodynamic properties of VRC01 suggest that administration will be needed every four weeks to maintain the concentration that is expected to be protective. Even if these administrations are timed to coincide with routine infant immunization schedules as much as possible, additional medical visits would be required, putting additional burden on families and health care systems. The situation is further complicated by the fact that many of the infants who would benefit most from this intervention live in communities with limited access to routine health care. The first translation efforts are focusing on proof-of-concept studies, but for optimal implementation, final products should be effective with less frequent administration. Fortunately, new approaches are being developed to extend the half-lives of monoclonal antibodies *in vivo* [29].

## Future Goals and Directions

Eliminating pediatric HIV infections worldwide is an achievable goal that needs to be approached from multiple directions. Improving delivery of proven interventions is critical and provides immediate return on investment. At the same time, the public health community should not become complacent in thinking that ARV drugs will provide adequate protection in all scenarios. Researchers need to actively explore new approaches that will complement and improve those that already exist. The discovery and isolation of extremely potent bNAbs present an opportunity that should be explored. Clinical trials are currently being planned that will provide valuable information about both the scientific and the logistical feasibility of bringing these discoveries into clinical use.

**Five Key Papers in the Field**

2013 UNAIDS progress report [2]. The report shows current progress in reducing MTCT in 21 priority countries.

Kesho Bora Study Group, de Vincenzi I (2011) [26]. The study showed excellent efficacy of triple-drug therapy for PMTCT, but at the same time showed that, even with this intervention, there is still a 5–6% transmission rate in the breastfeeding population.

Walker et al. (2009) [11]. The paper was the first in a series of papers from multiple laboratories describing isolation of the next generation of broad and potent neutralizing antibodies against HIV-1.

Wu et al. (2010) [13]. The authors used a specially designed envelope protein to isolate multiple broadly neutralizing antibodies, including VRC01.

Klein et al. (2013) [15]. The review discusses the potential applications of monoclonal antibodies in HIV treatment and prevention.

## Abbreviations

ANC: antenatal care
ARV: antiretroviral
bNAbs: broadly neutralizing antibodies
Ig: immunoglobulin
mAb: monoclonal antibodies
MTCT: mother-to-child transmission
PMTCT: prevent mother-to-child HIV transmission
RSV: respiratory syncytial virus
WHO: World Health Organization
